# Greywater Characteristics, Treatment Systems, Reuse Strategies and User Perception—a Review

**DOI:** 10.1007/s11270-018-3909-8

**Published:** 2018-07-16

**Authors:** Michael Oteng-Peprah, Mike Agbesi Acheampong, Nanne K. deVries

**Affiliations:** 1 Department of Health Promotion, Faculty of Health Medicine and Life Sciences, University of Maastricht, Peter Debyplein 1, 6229 HA Maastricht, The Netherlands; 2 Department of Chemistry, University of Cape Coast, Cape Coast, Ghana; 3 Department of Chemical Engineering, School of Engineering, Kumasi Technical University, Kumasi, Ghana

**Keywords:** Greywater, Reuse, Natural media, Treatment systems, User perception

## Abstract

This paper presents a literature review of the quality of greywater generated in different, especially developing, countries, constituents found in greywater, some treatment systems, natural materials for treatment, some reuse strategies and public perception regarding greywater reuse. The review shows that generation rates are mostly influenced by lifestyle, types of fixtures used and climatic conditions. Contaminants found in greywater are largely associated with the type of detergent used and influenced by other household practices. Many of the treatment systems reviewed were unable to provide total treatment as each system has its unique strength in removing a group of targeted pollutants. The review revealed that some naturally occurring materials such as *Moringa oleifera*, sawdust, can be used to remove targeted pollutants in greywater. The study further showed that user perceptions towards greywater treatment and reuse were only favourable towards non-potable purposes, mostly due to perceived contamination or lack of trust in the level of treatment offered by the treatment system.

## Introduction

The total volume of freshwater on Earth far outweighs the human demands. Out of the overall water resources on Earth, about 97% can be found in the oceans while the remaining 3% remains available for direct exploitation; however, out of this 3%, the quantity of water that is available for use by humans is estimated at one-hundredth (Eakin and Sharman 2010; Gleick 1993). Uneven distribution of water in both time and space sways the use of water to other geographical areas depriving others of this resource. Biological survival remains one of the key factors of water use with its associated use also for household needs and for food production and other developmental needs. Many parts of the world are hit by acute water shortage, over-exploitation of water resources leading to gradual destruction of these water resources and high levels of freshwater pollution resulting from anthropogenic factors. Currently, it has been estimated that about 800 million people live under a threshold of water stress and this number is expected to reach 3 billion in 2025 (Qureshi and Hanjra 2010; UNDP 2017). Due to urbanization, industrialization and population growth, the demand for water is evident; however, will the available water resources meet the ever-growing needs in a sustainable manner? Where will the extra water that is required to sustain human activities come from? This question calls for interventions and strategies that will help address these concerns. This is where a cursory look at greywater reuse is worthwhile.

**Fig. 1:**
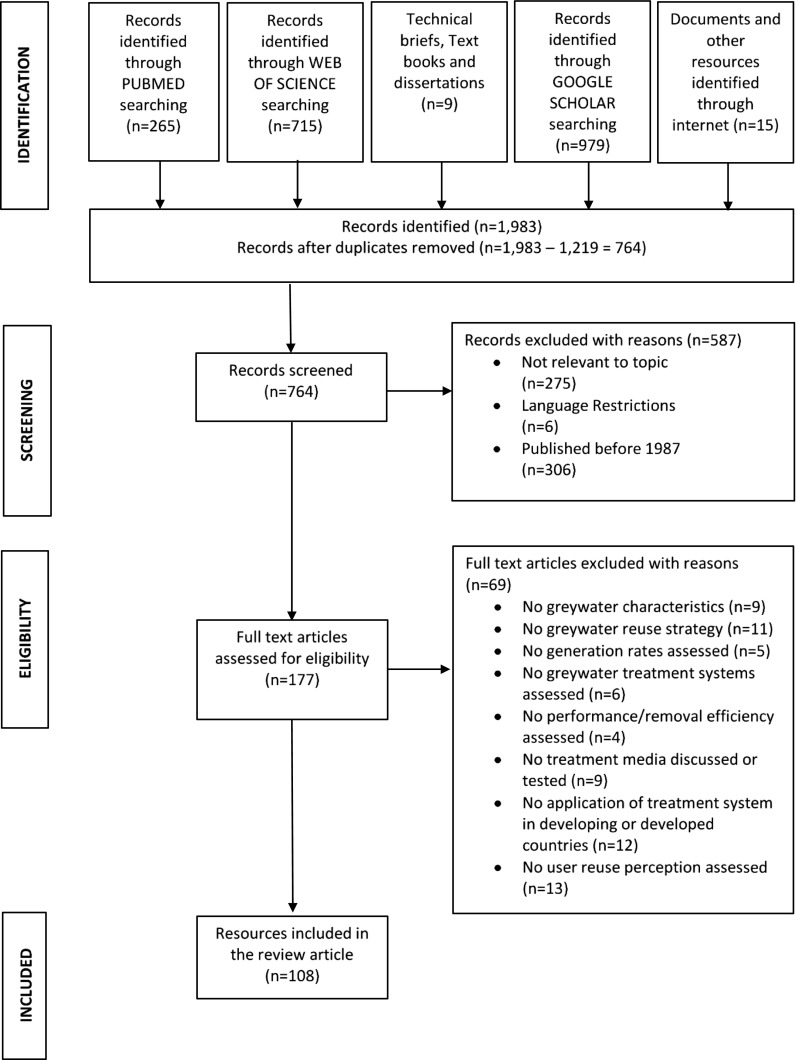
Methodological framework

Greywater is defined as wastewater without any contributions from toilet water (Casanova et al. 2001; Ledin et al. 2001; Ottoson and Stenstrom 2003). It is considered high volume, low strength wastewater with high potential for reuse and application. The composition of greywater is varied and depends on the lifestyle, fixtures and climatic conditions (Abedin and Rakib 2013; do Couto et al. 2013; Katukiza et al. 2014). Reuse of greywater has been an old practice, and it is still being done in areas that are water stressed. This practice if given the needed attention can help reduce the over-reliance on freshwater resources and reduce the pollution caused by discharge of untreated greywater into freshwater resources. It can also be a supplementary source to existing water sources in areas where there is acute water crisis or in arid climatic regions. Recycled greywater can be used for different water-demanding activities including potable and non-potable uses such as toilet flushing and agriculture. The major concerns with greywater reuse have been issues with public health perceptions and inappropriate technology for the reuse option (Vigneswaran and Sundaravadivel 2004). Many researchers have studied characteristics of greywater with respect to fixtures, life style patterns and type of settlement (Alsulaili and Hamoda 2015; do Couto et al. 2013; Katukiza et al. 2014). However, the aim of this study is to assess the performance of greywater treatment system, to further review greywater reuse perceptions, to identify gaps of greywater systems with emphasis on developing countries and to identify scope for further research. This review used resources from peer reviewed journals, documents from the internet and text books. The methodological framework that guided this review is presented in Fig. 1.

## Greywater Quantity

Greywater reuse has been considered as a reliable method of ensuring water security as compared to other methods of water capture such as rainwater harvesting which is dependent on hydrological conditions. The amount of greywater produced in a household can vary greatly ranging from as low as 15 L per person per day for poor areas to several hundred per person per day. Factors that account for such huge disparities are mostly attributed to geographical location, lifestyle, climatic conditions, type of infrastructure, culture and habits, among others. Greywater accounts for up to 75% of the wastewater volume produced by households, and this can increase to about 90% if dry toilets are used (Hernandez Leal et al. 2010). It has also been estimated that greywater produced accounts for about 69% of domestic water consumption (Jamrah et al. 2011). Table 1 presents different greywater generation rates in some reported studies in different countries.

**Table 1:** Greywater generation rates in different studies

Location	Generation (/Lc/day)	Reference
Africa and Middle East	14–161	Al-Hamaiedeh and Bino (2010); Halalsheh et al. (2008); Morel and Diener (2006)
Asia	72–225	Morel and Diener (2006)
Gauteng, South Africa	20	Adendorff and Stimie (2005)
Jordan	50	Faraqui and Al-Jayyousi (2002)
Mali	30	Alderlieste and Langeveld (2005)
Muscat, Oman	151	Jamrah et al. (2008)
Nepal	72	Shresta (1999)
Stockholm	65	Ottoson and Stenstrom (2003)
Tucson Arizona, USA	123	Casanova et al. (2001)
Vietnam	80–110	Busser et al. (2006)

## Greywater Composition

The composition of greywater varies, and it is largely a reflection of the lifestyle and the type and choice of chemicals used for laundry, cleaning and bathing. The quality of the water supply and the type of distribution network also affect the characteristics of greywater. There will be significant variations in the composition of greywater in both place and time which may be due to variations in water usage in relation to the discharged quantity. The composition may also be affected by chemical and biological degradations of some compounds within the transportation and storage network**.** Generally, greywater contains high concentrations of easily biodegradable organic materials and some basic constituents which are largely generated from households. These include nutrients such as nitrates and all its derivatives, phosphorus and its derivatives, but others include xenobiotic organic compounds (XOCs) (Fatta-Kassinos et al. 2011) and biological microbes such as faecal coliforms, salmonella and general hydrochemical constituents. Recent studies have however found pharmaceuticals, health and beauty products, aerosols, pigments (Eriksson et al. 2003) and toxic heavy metals such as Pb, Ni Cd, Cu, Hg and Cr (Aonghusa and Gray 2002; Eriksson et al. 2010) in appreciable concentrations in greywater. The presence of these contaminants in greywater is an indication of the gradual increase in the level of complexity in the composition of greywater.

## Physical Constituents

These are constituents that are associated with the physical appearance of greywater and include temperature, turbidity, electrical conductivity and suspended solids, among others. Greywater normally has temperature range of between 18 and 35 °C, and the rather high temperature may be originating from warm water used for personal hygiene and cooking activities. These high temperatures may favour microbiological growth which is undesirable and may also cause precipitation of certain carbonates such as CaCO_3_ and other inorganic salts which become less soluble at high temperatures. The concentration of total suspended solids in greywater can range within 190–537 mg/L as has been reported (Edwin et al. 2014; Oteng-Peprah et al. 2018). Greywater with much of the water originating from the kitchen and laundry accounts for the relatively high values of total suspended solids (TSS), and this may be due to washing of clothes, shoes, vegetables, fruits, tubers and many others which may contain sand, clay and other materials that could increase TSS. The ranges recorded for electrical conductivity in greywater is between 14 and 3000 μS/cm (Ciabatti et al. 2009; Prathapar et al. 2005). Groundwater sources and water scarce areas are mostly associated with high electrical conductivity due to dissolved materials. Poor or old plumbing materials also contribute to the increase in electrical conductivity due to leaching into greywater sources. The range of turbidity recorded for greywater is between 19 and 444 NTU, and it is mostly influenced by the water use activities. Greywater that has most of its sources originating from the kitchen and laundry is expected to become more turbid due to the presence of suspended matter.

## Chemical Contaminants

To identify the different chemical constituents in greywater, it is important to understand the sources of contaminants. Significant chemical constituents in greywater are from chemicals used for cleaning, cooking and bathing purposes. The pH in greywater to a large extent depends on the pH and alkalinity in the water supply and normally is within the range of 5–9. Greywater with most of its sources originating from the laundry will generally exhibit high pH due to the presence of alkaline materials used in detergents. The major chemical constituents found in greywater which is generated as a result of cleaning or washing activities are surfactant. These surfactants serve as the main active agent in most cleaning products. They can be either cationic or anionic in nature with a majority of cleaning and laundry products being anionic (Jakobi and Lohr 1987). Cationic surfactants are generally salt based, and they constitute a source of ammonium in the greywater. Other constituents found in greywater also include nitrates and phosphate which are reportedly from ammonium and cationic surfactants and laundry disinfectants respectively (Eriksson et al. 2002). Other constituents such as sodium which is also from cooking and preservation activities in the kitchen can also be found in appreciable levels. Sodium-based soaps also contribute significant quantity of sodium into greywater. Other additives such as builders control water hardness in detergents and also serve as the main source of phosphate contaminant in greywater (Lange 1994). Nutrients such as N and P are associated with kitchen and laundry activities. Greywater sources with high nutrients concentrations are mostly made up of a high fraction of kitchen and laundry sources (Boyjoo et al. 2013). Kitchen waste are the primary source of nitrogen in greywater and range between 4 and 74 mg/L while washing detergents are the primary source of phosphates found in grey water which also range between 4 and 14 mg/L (Boyjoo et al. 2013).

The conventional wastewater parameters such as biochemical oxygen demand (BOD_5_) and chemical oxygen demand (COD) always show a dominance of COD over BOD_5_. The biodegradability of greywater is determined by the BOD_5_/COD ratios. The ratio determines the ease with which bacteria can decompose the organic matter in the greywater. Mostly, all types of greywater show good biodegradability in terms of the BOD_5_/COD ratios (Li et al. 2009). The average BOD_5_/COD ratios in greywater have ranged between 0.31 and 0.71 which is an indication that almost half of the organic matter in greywater is biodegradable (Halalsheh et al. 2008). However, other studies have recorded ratios as high as 4:1 (Boyjoo et al. 2013). The dominance of COD to BOD_5_ has largely been due to the presence of XOCs that increases COD. XOCs are synthetic organic compounds that are present in household chemicals and pharmaceuticals such as bleaches, surfactants, softeners and builders and beauty products. XOCs can also be formed by partial modification of chemicals in chemical or biological treatment of greywater (Fatta-Kassinos et al. 2011). XOCs are recalcitrant to conventional treatment protocols and can easily accumulate in plants and animals and subsequently pose risks to the natural environment (Fatta-Kassinos et al. 2011). Eriksson et al. (2002) identified 900 potential XOCs in greywater solely based on the ingredients of different cosmetics and detergents in Denmark. Le-Minh et al. (2010) identified the presence of antibiotics in greywater which may lead to proliferation of resistant bacteria strains. Revitt et al. (2011) also identified the presence of benzene and 4-nitrophenol in greywater in appreciable concentrations. Other hazardous substances such as brominated flame retardants, polycyclic aromatic hydrocarbons, monocyclic aromatics, triclosans and phthalates have been identified in greywater (Palmquist and Hanaeus 2005). Table 2 presents some selected physicochemical parameters of greywater with their concentrations in some selected high- and low-income countries.

**Table 2:** Physicochemical characteristic of greywater s in low- and high-income countries

Parameter	Low-income countries				High-income countries			
	India^a^	Pakistan^b^	Niger^c^	Yemen^d^	USA^e^	UK^f^	Spain^g^	Germany^h^
pH	7.3-8.1	6.2	6.9	6	6.4	6.6–7.6	7.6	7.6
Turbidity (NTU)	–	–	85	619	31.1	26.5–164	20	29
EC (μS/m)	–	–	–	–	23	32.7	–	64.5
TSS (mg/L)	100–283	155	–	511	17	37–153	32	–
TDS (mg/L)	573	102	–	–	171	–	–	–
BOD_5_ (mg/L)	100–188	56	106	518	86	39–155	–	59
COD (mg/L)	250–375	146	–	2000	–	96–587	151–177	109
Cl (mg/L)	53	–	–	–	–	–	–	–
Oil and grease (mg/L)	7	–	–	–	–	–	–	–
Nitrate (mg/L)	0.67	–	–	98	–	3.9	–	–
T. Nitrate (mg/L)	–	–	–	–	13.5	4.6–10.4	10–11	15.2
T. Phosp (mg/L)	0.012	–	–	–	4	0.4–0.9	–	1.6
FC (CFU)	–	–	–	1.9	–	–	–	1.4 × 10^5^
*E. coli* (CFU)	–	–	–	–	5.4 × 10^5^	10–3.9 × 10^5^	–	–
Ca (mg/L)	0.13	–	–	–	–	–	–	–
Mg (mg/L)	0.11	–	–	–	–	–	–	–
Na (mg/L)	32–50	–	–	–	–	–	–	–

^a^Parjane and Sane (2011)

^b^Pathan et al. (2011)

^c^Hu et al. (2011)

^d^Al-Mughalles et al. (2012)

^e^Jokerst et al. (2011)

^f^Birks and Hills (2007); Pidou et al. (2008)

^g^March and Gual (2007); March et al. (2004)

^h^Merz et al. (2007)

## Biological Characteristics

Greywater contains microorganisms such as bacteria, protozoa and helminths which are introduced into it by body contact. Inappropriate food handling in the kitchen and direct handling of contaminated food have been identified as sources of enteric pathogenic bacteria such as *Salmonella* and *Campylobacter* into greywater (Maimon et al. 2014; Ottoson and Stenstrom 2003). Faecal contamination is also common in greywater and is largely associated with poor personal hygiene and disposal of greywater which contains washed nappies. Pathogenic *Escherichia coli* and enteric viruses have been detected in greywater with majority of the water originating from laundry sources during a microbial monitoring programme in Melbourne Australia (O’Toole et al. 2012). In this study, 18% of samples contained enteric viruses, 7% enterovirus and 11% of *E. coli*. The most common indicators used to assess faecal contamination are coliform bacteria and *E. coli*. Studies conducted by Eriksson et al. (2002) and Ottoson and Stenstrom (2003) revealed a large collection of excreta-related pathogens associated with greywater**.** Other studies have further identified a number of pathogens in greywater, and these are *Pseudomonas* (Benami et al. 2015a; Khalaphallah and Andres 2012), *Legionella* (Birks et al. 2004), *Giardia* (Birks et al. 2004; Birks and Hills 2007), *Cryptosporidium* (Birks et al. 2004) and *Staphylococcus aureus* (Benami et al. 2015a; Kim et al. 2009; Maimon et al. 2014; Shoults and Ashbolt 2017) in greywater. Table 3 presents some selected biological parameters with their concentrations as reported in other studies.

**Table 3:** Biological characteristic of greywater in low- and high-income countries

Name of microbe	Concentration	Source
Total coliforms (counts/100 mL)	1.2 × 10^3^–8.2 × 10^8^	Alsulaili et al. (2017); Dwumfour-Asare et al. (2017); Mandal et al. (2011); Masi et al. (2010); Oteng-Peprah et al. (2018)
*E. coli*	Up to 6.5 × 10^6^	Atanasova et al. (2017); Friedler et al. (2006a); Khalaphallah and Andres (2012); Kim et al. (2009); Oteng-Peprah et al. (2018); Paulo et al. (2009)
Faecal coliforms	Up to 1 × 10^6^	Halalsheh et al. (2008); Mandal et al. (2011); Masi et al. (2010)
*Pseudomonas aeruginosa*	1.4 × 10^4^	Benami et al. (2015a); Khalaphallah and Andres (2012)
*Staphylococcus aureus*	1.2 × 10^2^–1.8 × 10^3^	Benami et al. (2015b); Kim et al. (2009); Maimon et al. (2014); Shoults and Ashbolt (2017)
*Salmonella typhi*	5.4 × 10^3^	Kim et al. (2009)
*Salmonella* spp.	3.1 × 10^3^	Oteng-Peprah et al. (2018)

## Treatment Systems

Management of greywater graduates from simple to extremely complex when the necessary strategies and technology is not in place or not properly implemented. Many developed countries have however implemented from simple to advanced methods of handling, managing and treating greywater with some countries recycling the greywater for both potable and non-potable uses. Treatment systems have been used to reduce the level of contamination in greywater before reuse or final disposal. They are contaminant-specific, and each is applied along the conventional wastewater treatment sequence (pre-treatment, primary, secondary and tertiary treatment). Each of these systems adopts either a physicochemical or biological means of treatment. Physicochemical methods adopt physical and/or chemical methods of treatment including filtration, adsorption and reverse osmosis, among others. Biological treatment methods adopt a combination of microbes, sunlight and oxygen manipulation; examples of such systems include activated sludge systems, trickling filters, waste stabilization ponds, rotating biological contactors and many others. The widely used systems have mostly been filtration, rotating biological contactors, membrane bioreactors, constructed wetlands and upflow anaerobic sludge blankets (UASBs). These systems have found their application in addressing the emerging greywater pollution experienced in most developing countries. This review therefore discusses the performance of these systems.

## Filtration

Filtration involves removal of particulate matter which is not removed by preceding processes. In filtration systems, both physical and biological processes remove solids; however, this review considers only physical removal of solids because that is the method adopted in most greywater treatment schemes. Filtration media could be in the form of sand, gravel, fine mesh and many others. Gross et al. (2007) studied the performance of a filtration system in greywater treatment using pebbles of 2 cm thick placed over drain holes and followed by a 12-cm middle layer consisting of 12 cm of plastic filter media and finally topped by 4 cm thick layer of peat. Dalahmeh et al. (2012) also studied the performance of a filtration system using pine bark, activated charcoal, polyurethane foam and sand as filter media in treating greywater. The performance of a coarse filtration system followed by slow sand filtration with a hydraulic retention time of 8 and 24 h respectively was studied by Finley et al. (2009). Parjane and Sane (2011) used coconut shell, coarse sawdust, charcoal, bricks and sand as filter materials to assess the performance of greywater treatment. A four-barrel filtration unit has been used to investigate greywater treatment by Al-Hamaiedeh and Bino (2010). These barrels were arranged in series, and the first three were loaded with gravels of 2–3 cm diameter. The final barrel was used to collect the treated effluent for irrigation. Gross (2008) adopted a hybrid filtration system utilizing a 130-μm net filtration, a tuff filter, a sand filter and followed by electrolysis in Israel. Zuma et al. (2009) used a mulch tower system to treat greywater in South Africa. This was constructed by using mulch, coarse sand, fine and coarse gravel. This was contained in a 650-mm high plastic column of 150 mm diameter with a stainless-steel sieve mesh placed on top to remove big particles. From the reviewed filtration systems, only bark filters were able to meet the pH criteria for reuse. More so, only the bark and charcoal filters could meet the BOD_5_ regulatory standard for reuse. Removal rates of total phosphorous were high in bark, charcoal and sand filters. The performance of filtration systems discussed is presented in Table 4.

**Table 4:** Treatment efficiencies of some selected greywater treatment systems

Parameter	Filtration^a^	Wetlands^b^	SBR^c^	RBC^d^	MBR^e^	UASB^f^
Turbidity (NTU)	–	–	–	–	98–99%	–
EC (uS/m)	–	–	–	–	–	–
TSS (mg/L)	53–93%	90–98%	–	9–12%	Up to 100%	–
TDS (mg/L)	–	–	–	–	–	–
BOD_5_ (mg/L)	89–98%	Up to 99%	90–98%	27–53%	93–97%	Up to 67%
COD (mg/L)	37–94%	81–82%	90–98%	21–61%	86–99%	38–79%
Cl (mg/L)	–	92–94%	–	–	–	–
Oil and grease (mg/L)	Up to 97%	Up to 95.45	–	–	–	83.7%
Nitrate (mg/L)	17–73%	–	–	–	6–72%	–
T. Nitrate (mg/L)	5–98%	26–82	80%	–	52–63%	24 to 58%
T. Phosp (mg/L)	Up to 100%	Up to 71%	–	–	Up to 19%	10 to 39%
FC (CFU)	–	–		88.5–99.9%	Up to 99%	–
*E. coli* (CFU)	Up to 100%	–		88.5–99.9%	–	–
Ca (mg/L)	Up to 100%	–	–	–	–	–
Mg (mg/L)	Up to 100%	–	–	–	–	–
Na (mg/L)	47%	–	–	–	–	–

^a^Al-Hamaiedeh and Bino (2010); Dalahmeh et al. (2012); Finley et al. (2009); Gross (2008); Parjane and Sane (2011); Zuma et al. (2009)

^b^Gross (2008); Gross et al. (2007); Travis et al. (2010)

^c^Hernandez Leal et al. (2010); Krishnan et al. (2008); Lamine et al. (2007); Scheumann and Kraume (2009)

^d^Friedler et al. (2011); Gilboa and Friedler (2008); Pathan et al. (2011)

^e^Atanasova et al. (2017); Huelgas and Funamizu (2010); Jong et al. (2010); Merz et al. (2007)

^f^Abdel-Shafy et al. (2015); Elmitwalli et al. (2007); Hernandez Leal et al. (2010)

## Constructed Wetland

Constructed wetland (CW) is an artificial wetland constructed utilizing ecological technology to mimic conditions that occur in a natural wetland. The technology adopts special flora and fauna, soil and microorganisms to remove pollutants of interest. They are normally classified under three main types namely subsurface flow, surface flow and floating treatment wetland. The subsurface flow systems have been the most widely used constructed wetlands, and they come in two main technologies, vertical flow constructed wetland (RVFCW) and horizontal flow constructed wetland (HFCW). Each removes contaminants by a combination of physical, chemical and biological processes, and the treatment efficiency depends on factors such as loading rate and the availability of electron acceptors (Halalsheh et al. 2008). They have high potential of removing BOD_5_, suspended solids and some heavy metals such as Pb, Zn and Fe, among others. The performance of a RVFCW was studied, and it was observed that removal of ammonia nitrate was very low as compared to other systems (Gross et al. 2007; Travis et al. 2010). Gross (2008) also investigated the performance of HFCW in greywater treatment and observed that the quality of effluent improved if there was a pretreatment of the greywater**.** In this study, the average retention time was about 30 h and it was realized that electrical conductivity increased from 170 to 190 mS/m, T-N was reduced from 31 to 23 mg/L and T-P was also reduced from 48 to 46 mg/L representing 25.8 and 4.2% respectively. One major advantage of CW is its ability to run on its own without the attention of an operator. However, its removal rates for Na, Ca and Mg are relatively low and it also leads to increases in electrical conductivity (EC) which might be due to the dissolution of organic matter in the treated water leading to increase in the total dissolved solids (TDS) and subsequently affecting the EC. They are also unable to remove some microbiological agents such as *E. coli* and helminth eggs and as such will require further treatment if the objective of the treatment is reused. However, CW can produce effluents with BOD_5_ and TSS meeting the regulatory limits. The removal efficiencies of this discussed CW are presented in Table 4.

## Rotating Biological Contactors

Rotating biological contactors (RBCs) are fixed bed reactors consisting of rotating disks and mounted on a horizontal shaft. They are partially submerged and rotated as wastewater flows through. The microbes that do the treatment are alternatively exposed to the atmosphere allowing both aeration and assimilation of dissolved organic pollutants and nutrients for degradation. Pathan et al. (2011) studied the performance of a single-stage RBC on greywater in Pakistan. The RBC was made of plastic sheets and the disks from textured plastic. The greywater was kept in the system for a specified period of time while the rotating discs were submerged up to 40% in the greywater**.** Friedler et al. (2011) studied the potential of RBC to remove indicator bacteria (faecal coliforms, heterotrophic bacteria) and specific pathogens (*Pseudomonas aeruginosa* sp., *Staphylococcus aureus* sp.). The study concluded that RBC removed 88.5–99.9% of all four bacteria groups. Gilboa and Friedler (2008) studied the performance of RBC on removal of faecal coliforms (FC), *Staphylococcus Aureus* sp., *Pseudomonas aeruginosa* sp. and *Clostridium perfringens* sp. in greywater using RBC followed by sedimentation. The study concluded that the system removed up to 99% of all these microorganisms which were in the greywater. RBC systems perform well with respect to pH, BOD_5_, COD, reduced microbial loads and produced effluents that meet discharge guidelines. Performance of the system is presented in Table 4.

## Sequencing Batch Reactor

A sequencing batch reactor (SBR) is a type of activated sludge process used for wastewater treatment. All the treatment process takes place in batches in the reactor tank. The batch is sequenced through a series of treatment stages and performs equalization, biological treatment and secondary clarification in a single tank using a time-controlled sequence. Lamine et al. (2007) conducted a study on greywater treatment using SBR in a student house. This study assessed the performance of treatment by varying the hydraulic retention times (HRTs), and it revealed an effect on nitrification with the varying HRTs**.** A similar study by Scheumann and Kraume (2009) also used a pilot scale SBR by varying the retention time and observed the removal of COD, NH_4_-N and TN was sufficient to meet discharge reuse guidelines; however, there was nitrification in this study as also reported by Lamine et al. (2007). In this study, feedstock concentration of COD 250 mg/L, NH4-N 11.9 mg/L and TN = 17.1 were reduced to 18.9, 4.1 and 0.37 mg/L respectively, all being below the mandatory values for reuse applications. Krishnan et al. (2008) investigated the performance of greywater treatment from residential houses in Malaysia on a square bottom SBR at fixed HRT. An SBR has also been used in a demonstration project in the Netherlands to treat greywater from 32 houses (Hernandez Leal et al. 2010). SBRs have removal efficiencies of up to 98% for BOD_5_ and COD, up to 80% TN and up to 99% for NH_4_-N**.** The HRT has been found to be a limiting factor in the performance of SBRs since difference HRTs result in different effluent qualities as is shown in the different studies (Lamine et al. 2007; Scheumann and Kraume 2009). The performance of this system is presented in Table 4.

## Membrane Bioreactor

A membrane bioreactor (MBR) is a perm-selective process integrated with a biological process for treating greywater. It works on a combination of biological, microfiltration and ultrafiltration systems to achieve treatment. It is an appropriate solution that can be used for greywater treatment and reused in densely urbanized areas, where space has high value, due to its compact size. Atanasova et al. (2017) studied the performance of an MBR on greywater treatment in a hotel in Spain. The removal efficiency for COD ranged from 80 to 95%, where COD concentration in the effluent was below the quantification limit 30 mg/L based on the Spanish legislation for water reuse. Ammonia and TN removal were on average at high level 80.5 and 85.1% respectively. The treatment performance of an MBR made up of an ultrafitration membrane was also studied by Merz et al. (2007) on greywater from a sports complex in Morocco. Huelgas and Funamizu (2010) studied the treatment of greywater using a laboratory scale MBR under varying pressure. Jong et al. (2010) also used anaerobic-anoxic-oxic MBR to treat greywater in Korea with microfilter of pore size 0.45 μm. These systems could achieve very good effluent which meets regulatory standards for reuse. A nominal pore size of 0.1 μm has been found to remove faecal coliforms. Performance of this system is presented in Table 4.

## Upflow Anaerobic Sludge Blanket

The upflow anaerobic sludge blanket (UASB) has remained one of the most widely used wastewater treatment system for various types of wastewater streams. It works on an anaerobic process and retains a high concentration of active suspended biomass and produces better settleable sludge than other treatment systems. Greywater from 32 houses in the Netherlands was treated using this system (Hernandez Leal et al. 2010). Elmitwalli et al. (2007) also used this system to study the treatment of greywater in Lubeck Germany by varying the retention time. Abdel-Shafy et al. (2015) investigated the efficiency of UASB in greywater treatment for unrestricted use in Egypt. The raw greywater characteristics with average concentrations of 95, 392, 298, 10.45, 0.4, 118.5 and 28 mg/L for TSS, COD, BOD_5_, TP, nitrates, oil and grease and TKN respectively were treated in a UASB. After treatment, the effluent concentration was 76.65, 165.4 96.85 and 19.31 mg/L for TSS, COD, BOD_5_ and oil and grease respectively. This represents removal efficiencies of 19.3% for TSS, 57.8% for COD, 67.5% for BOD_5_ and 83% for oil and grease. UASBs perform better when they are integrated with other systems. The performance of the system is presented in Table 4.

## Naturally Occurring Greywater Treatment Media

These are naturally occurring materials that have been applied as either standalone media or used as a complementary medium in some of the available conventional greywater treatment systems. Many researchers have studied the effect of treatment offered by these media, and their performance is listed in Table 5. Unlike other conventional treatment systems, these media are used for removing targeted contaminants and their mode of treatment is either by adsorption, filtration or coagulation.

**Table 5:** Naturally occurring materials for greywater treatment

Type of material	Target pollutant removal	Percentage removal (%)	Mode of removal	Source
Activated carbon	BOD_5_ COD TN TP	97 94 98 91	Adsorption	Sahar et al. (2012)
Activated charcoal	EC BOD_5_	12 97	Adsorption	Dalahmeh et al. (2012)
Peat moss and lime pebbles	COD BOD_5_ *E. coli*	90 95 100	Filtration	
Pine bark	BOD_5_ COD TN TP	98 74 19 97	Adsorption	Sahar et al. (2012)
Moringa oleifera	COD	64	Coagulation	Bhuptawat et al. (2007)
	Turbidity Conductivity BOD_5_	98 11 12		Hendrawati et al. (2016)
	Turbidity TSS	96 88		Effendi et al. (2015)
Sawdust	TSS TDS O&G COD	83 70 97 82	Filtration	Parjane and Sane (2011)

## Reuse Strategies

A number of greywater treatment and reuse schemes have been implemented across the globe using both conventional and hybrid systems. Most of these systems have been developed as an environmental intervention measure and have since been operational while some have had their own challenges from both the technical and public point of view. Table 6 presents some examples of successful application of greywater treatment and reuse schemes in some countries.

**Table 6:** Greywater reuse strategies in some developing countries

Location	System used	Application	Performance	Source
Auroville, India	Reed beds and irrigation beds using banana	Treats greywater from a student dormitory		Chuck (2004)
Koulikoro, Mali	Vertical flow filter and greywater garden	Treats greywater generated by a community and the treated greywater is used in subsurface irrigation of fruits and vegetables		GTZ (2005)
Mexico	Bioreactor and mulch bed	Treats greywater for a rehabilitation centre for children		
Djenne, Mali	Infiltration trench	Was intended to stop the unregulated discharge of greywater into the streets. Unsightly conditions ceased within the community because greywater was discharged into trenches		Alderlieste and Langeveld (2005)
Gauteng, South Africa	Tower garden	Was intended to promote gardening due to proximity to water for irrigation and further empower the unemployed aged financially. Leafy vegetables are planted into the silts, and they are embraced by many people.		Adendorff and Stimie (2005)
Monteverde, Costa Rica	Constructed wetlands, Submerged flow reedbeds	Was intended to be used to treat greywater from single households to prevent discharge of greywater into the environment. Treated greywater was used to irrigate reeds which were an economic plant.		Dallas et al. (2004)
Kuching, Malaysia	Anaerobic filter, horizontal flow planted filter	An intervention to stop discharge of septic tank effluent directly into stormwater drains and subsequently into receiving water	Oil and grease 99% TSS 96% BOD_5_ 99% COD 95% NH_4_-N 94% T-P 88% T-N 76%	Chong (2005)
Billen, Palestine	Anaerobic upflow filters, aerobic filter	Intended to reduce frequency of desludging in a city which is water stressed	TSS 93–96% BOD_5_ 78–95% PO_4_-N 39–74% NO_3_-N 39–74%	Mahmoud et al. (2003)
Sri Lanka	Anaerobic filter, vertical-flow planted filter	Greywater treatment systems in some selected hotels and schools		Harindra (2001)
Kathmandu, Nepal	Vertical flow planted filter	A local responsive approach to solve problems of water scarcity in Nepal. The greywater treated is reused for other non-potable purposes while the impact of this system leads to significant savings in water expenditure.	TSS 97% BOD_5_ 98% COD 93% PO_4_-P 33% NH_4_-N 96%	Shrestha et al. (2001)
Monteverde, Costa Rica	Horizontal-flow planted filter	An intervention to stop haphazard discharge of greywater onto the streets and into streams. This caused unsightly conditions. After construction of this system, conditions improved.	BOD_5_ 99% NH_4_-N 95% PO_4_-P 84%	Dallas and Ho (2005)
Tufileh, Jordan	Automated greywater system	Optimization and validation of a system for reusing greywater in home gardens in Jordan		Al-Jayousi (2003)

## Greywater Reuse Perceptions

Public perception which is a social phenomenon can be seen as the difference between an absolute truth based on facts and virtual truth shaped by popular opinion (Conjucture 2017). In implementation of any project, public perception has been recognized as an integral factor in determining the success of the project. Many technically sound and environmentally friendly programs have failed because it was not accepted by the intended beneficiaries. Several studies have been conducted to assess public perception towards greywater reuse in different parts of the world using different strategies. These strategies include interviews, questionnaires, focus group discussions, informal discussions and other equally good social surveys. Most of these surveys identified clear support for the concept of greywater reuse as an environmentally sustainable method of protecting freshwater resources and pollution prevention. It has been reported by Dolnicar and Schafer (2006), Friedler et al. (2006b), Hurliman and McKay (2007), Kantanonleon et al. (2007) and Marks (2004) that the highest acceptability of greywater reuse schemes are for non-potable uses. Dolnicar and Schafer (2006) identified reduced levels of acceptance as the recycled water got closer to human contact. A similar study by Jeffery (2001) in the UK identified that people were more willing to use ‘own’ recycled greywater than to use recycled water from an unknown source. Alhumoud and Madzikanda (2010) identified that public support was greater for areas which are water stressed and areas with unreliable water supply. The results of a study by Adewumi et al. (2010) conducted in three universities in South Africa among students and staff concluded that the level of education and level of awareness contribute to the success of greywater reuse. Religious and cultural practices have been identified as factors that influence reuse programs. This is supported by another study by DeSena (2006) and Parkinson (2008) who identified misinformation, lack of knowledge or instinctive repugnance as accounting for objections in reuse programs.

## Potable Reuse Perceptions

The interruption and complete obstruction of many potable water reuse projects by stiff public opposition have been reported by DeSena (2006), Hurliman and Dolnicar (2013) and Meehan et al. (2013). This stiff opposition to potable reuse has been attributed to the close association of greywater with sewage which creates a phenomenon known as wisdom of repugnance. This phenomenon assumes that recycled water is associated with human waste; therefore, it renders it unpalatable from the public’s point of view. A study by Marks (2004) in three developed countries (Australia, the USA and the UK) identified low public support and acceptance for greywater reuse for potable purposes. The main barrier encountered in these studies were the associated perceived health risk of reusing recycled water in activities that involve direct contact with the user. Other studies identified language of the names given to recycled water as one of the obstacles affecting reuse schemes (Dolnicar and Saunders 2006). A study by Friedler et al. (2006b), Omerod and Scott (2013) and Russell et al. (2009) identified public trust arising out of a combination of technical and non-technical issues. The study identified strong public opposition to reuse projects, where there is little trust in the implementing body even in the face of the most advanced technology applicable. Currently, most of the research in this area is targeting determinants that increases acceptance of reuse programs.

## Conclusions

This study reviewed greywater characteristics, treatment systems, reuse strategies and perception of greywater reuse among users. It shows that there is a wide variation in greywater characteristics and volume generation rates which is largely dependent on the water use, lifestyle patterns and type of settlement. From the list of reviewed conventional treatment systems, filtration methods seem feasible and have the potential of integration with other systems to achieve target specific treatment. The study described different reuse strategies, most using discharged greywater for food production and landscaping while others have been used for poverty alleviation in irrigation farming.

The available technologies have been developed to treat or remove specific pollutants and not offer a full treatment of the greywater. Moreover, quality criteria differ for each type of reuse application, and greywater composition and generation rates vary greatly from one point to the other. It will therefore be prudent if systems are designed to target a specific reuse option taking into consideration the regional variability and complexities such that effluent from a treatment system will meet the required effluent guidelines. All the treatment systems reviewed were applicable on a large scale and cannot be applied at the household level. This in our view discourages local level participation in greywater reuse schemes. From the review, the potential of some natural materials to be used as media in greywater treatment systems also emerged. These natural materials are widely available in most developing countries, and their total integration into the conventional treatment systems should be explored. They can be used to design simple household level greywater treatment systems that target a certain reuse option and thereby increase local level participation.

Perception of greywater reuse has been closely related to the choice of reuse as most users will want to reuse greywater for activities that do not involve personal contact. In general, public perceptions are important to consider when implementing a certain method for a specified use. On the basis of this review, we conclude that to achieve effective greywater treatment and reuse, extensive contributions from technical and non-technical experts in many disciplines are called for. It also requires a comprehensive assessment of the greywater characteristics in order to choose an appropriate method or system of treatment. That notwithstanding, greywater treatment and reuse if embraced and enforced can lead to substantial decline in over-reliance on freshwater resources for non-potable uses.
